# Heterologous fibrin sealant derived from snake venom: from bench to bedside – an overview

**DOI:** 10.1186/s40409-017-0109-8

**Published:** 2017-04-04

**Authors:** Rui Seabra Ferreira, Luciana Curtolo de Barros, Luciana Patrícia Fernandes Abbade, Silvia Regina Catharino Sartori Barraviera, Maria Regina Cavariani Silvares, Leticia Gomes de Pontes, Lucilene Delazari dos Santos, Benedito Barraviera

**Affiliations:** 1 Graduate Program in Tropical Diseases, Botucatu Medical School, São Paulo State University (UNESP – Univ Estadual Paulista), Botucatu, SP Brazil; 2 Center for the Study of Venoms and Venomous Animals (CEVAP), São Paulo State University (UNESP – Univ Estadual Paulista), Botucatu, SP Brazil; 3 Department of Dermatology and Radiology, Botucatu Medical School, São Paulo State University (UNESP – Univ Estadual Paulista), Botucatu, SP Brazil; 4 CEVAP/UNESP, Avenida José Barbosa de Barros, 1780, Botucatu, SP CEP 18610-307 Brazil

**Keywords:** Fibrin sealant, Snake venom, Cryoprecipitate coagulum, Thrombin-like enzyme, Buffaloes

## Abstract

Hemostatic and adhesive agents date back to World War II, when homologous fibrin sealant came onto scene. Considering that infectious diseases can be transmitted via human blood, a new heterologous fibrin sealant was standardized in the 1990s. Its components were a serine protease (a thrombin-like enzyme) extracted from the venom of *Crotalus durissus terrificus* snakes and a fibrinogen-rich cryoprecipitate extracted from the blood of *Bubalus bubalis* buffaloes. This new bioproduct has been used as a coagulant, sealant, adhesive and recently as a candidate scaffold for mesenchymal stem cells and bone and cartilage repair. This review discusses the composition of a new heterologous fibrin sealant, and cites published articles related to its preclinical applications aiming at repairing nervous system traumas and regenerating bone marrow. Finally, we present an innovative safety trial I/II that found the product to be a safe and clinically promising candidate for treating chronic venous ulcers. A multicenter clinical trial, phase II/III, with a larger number of participants will be performed to prove the efficacy of an innovative biopharmaceutical product derived from animal venom.

**Electronic supplementary material:**

The online version of this article (doi:10.1186/s40409-017-0109-8) contains supplementary material, which is available to authorized users.

## Background

The first research studies on hemostatic agents and adhesives date back to World War II, when fibrin glue was proposed. In that time, a mixture of human fibrinogen and thrombin was applied to the affected aera. In 1970, given that the basic principles for extracting fibrinogen-rich cryoprecipitate and coagulation factors were already known, the concept of fibrin glue was reevaluated. Since that point, a new adhesive has been standardized with the following composition: fibrinogen-rich human cryoprecipitate, bovine thrombin and calcium chloride as the diluent. This sealant was successfully commercialized for years [1, 2].

In 1978, the U.S. Food and Drug Administration (FDA) suspended its commercialization due to the possibility of the transmission of infectious diseases, carried via products derived from human blood [3, 4].

In order to overcome these difficulties, in the 1990s the Center for the Study of Venoms and Venomous Animals (CEVAP) at São Paulo State University (UNESP) initiated studies to achieve the standardization of a new heterologous fibrin sealant (HFS). After several experiments, a new sealant was proposed, which was composed of a fibrinogen-rich cryoprecipitate extracted from the blood of the buffalo *Bubalus bubalis* in association with a serine protease (a thrombin-like enzyme) extracted from *Crotalus durissus terrificus* venom [5–8].

The active ingredient of this new heterologous fibrin sealant mimics the final step of the coagulation cascade. So, a thrombin-like enzyme acts upon the fibrinogen molecule, transforming it into fibrin monomers that polymerize in the presence of calcium to form a stable clot with adhesive, hemostatic and sealant effects [8, 9].

**Fig. 1:**
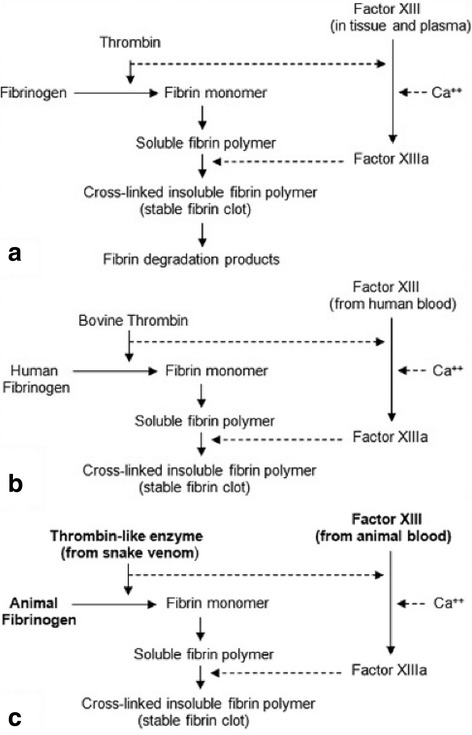
**a** The final common pathway of the human blood-clotting cascade. **b** The effect of bovine thrombin on human fibrinogen. **c** The effect of a serine protease (thrombin-like enzyme) extracted from snake venom on animal fibrinogen forming a stable fibrin polymer. Reprinted from “A new fibrin sealant from *Crotalus durissus terrificus* venom: applications in medicine” by LC Barros et al., *J Toxicol Environ Health B Crit Rev*, 2009, 12(8), 553–71 [9]. Copyright by Taylor & Francis LLC (http://www.tandfonline.com). Reprinted with permission

**Fig. 2:**
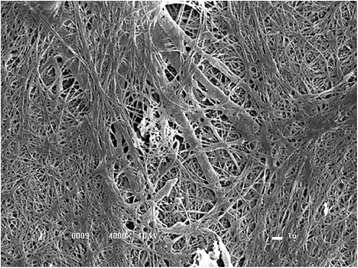
Stable fibrin network visualized in an electron microscope (4,000×). Reprinted from “A new fibrin sealant as a three-dimensional scaffold candidate for mesenchymal stem cells” by VPO Gasparotto et al., *Stem Cell Res Ther,* 2014, 5(3), 78 [10]. Copyright by VPO Gasparotto et al. Reprinted with permission

Figure 1 shows the blood-clotting cascade in three different pathways, with human thrombin, bovine thrombin and serine protease extracted from *Crotalus durissus terrificus* venom [9]. Figure 2 shows a stable network of fibrin formed from a mixture of animal cryoprecipitate with serine protease extracted from snake venom, observed by electron microscopy [10].

## Composition of the heterologous fibrin sealant

### Fraction I: serine protease (gyroxin)

#### Molecular structure

The composition of the venom from *Crotalus durissus terrificus* snakes is complex and constituted of enzymes, toxins and peptides. Since the 1980s, several authors have studied, isolated and purified serine proteases including gyroxin, a thrombin-like enzyme extracted from the venom of *Crotalus durissus terrificus* [11–15]. Electrophoretic analysis verified that this enzyme is a single-chain type, with an estimated molecular mass of 34 kDa and maximum stability at pH 8.0, and does not present alteration by freezing or thawing. Its maximum enzymatic activity occurs at pH 4.0, being resistant to treatment at 40 ° C for 15 min.

**Fig. 3:**
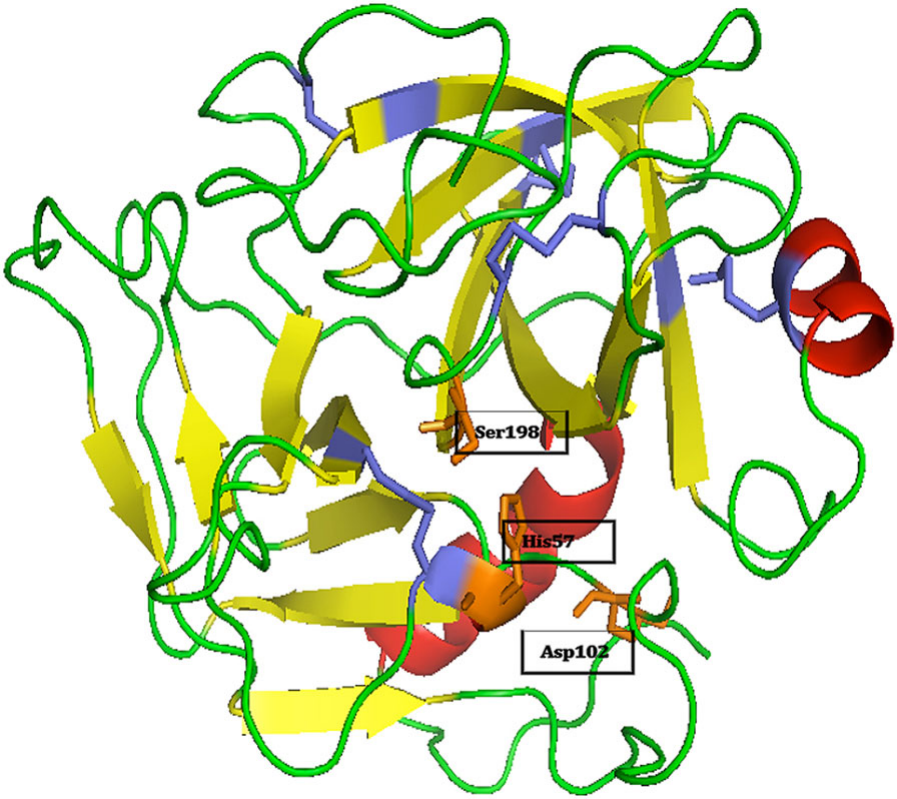
Theoretical molecular modeling of gyroxin accomplished using Modeller and PyMOL programs. This serine protease has two α-helix structures (*red*) containing the residues 146–152 and 215–227, two β-barris structures formed by six antiparallel sheets and loops (*green*), five disulfide bridges (*blue*) and a catalytic triad (*orange*)

Theoretical molecular modeling of this serine protease extracted from *Crotalus durissus terrificus* venom was accomplished via the program Modeller and visualization of the model by the program PyMOL. In this manner, Fig. 3 shows the structural model, which was revealed as a monomeric globular structure, presenting two α-helix structures (red) containing the residues 146–152 and 215–227, two β-barris structures formed by six antiparallel sheets and loops (green), five disulfide bridges (blue) and a catalytic triad (orange) [16, 17].

Due to its enzymatic activity, similar to thrombin, the serine protease acts on human and animal fibrinogen, cleaving the α-chain proximal to the N-terminal. The resultant fibrin monomers were polymerized in an intense stable network (Fig. 2) in contrast to that traditionally produced by thrombin.

#### Isolation and structural elucidation

**Fig. 4:**
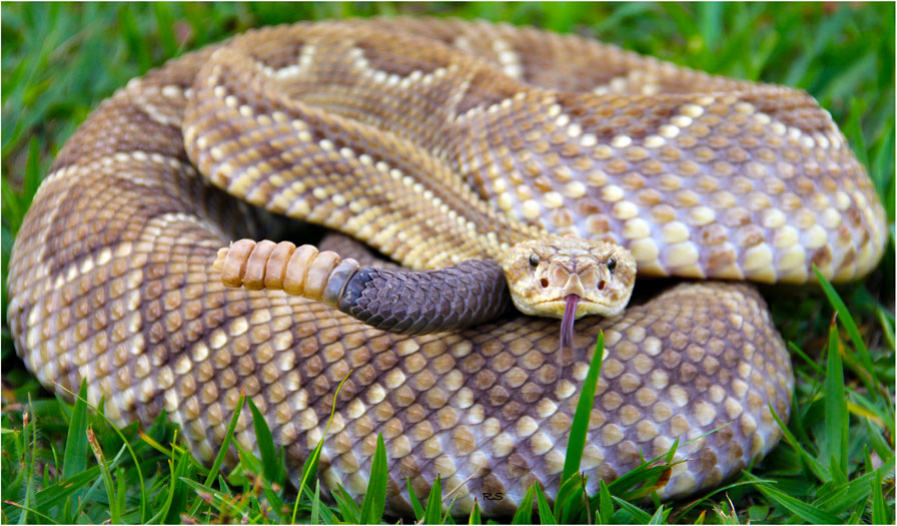
*Crotalus durissus terrificus* snake

Venom from *Crotalus durissus terrificus* snakes (Fig. 4) was milked at CEVAP and pooled according to good manufacturing practices (GMP). All the snakes are microchipped to ensure the traceability of the venom lots used in the composition of heterologous fibrin sealant. After filtration and lyophilization, the venoms are stored in the Venoms Bank of CEVAP.

**Fig. 5:**
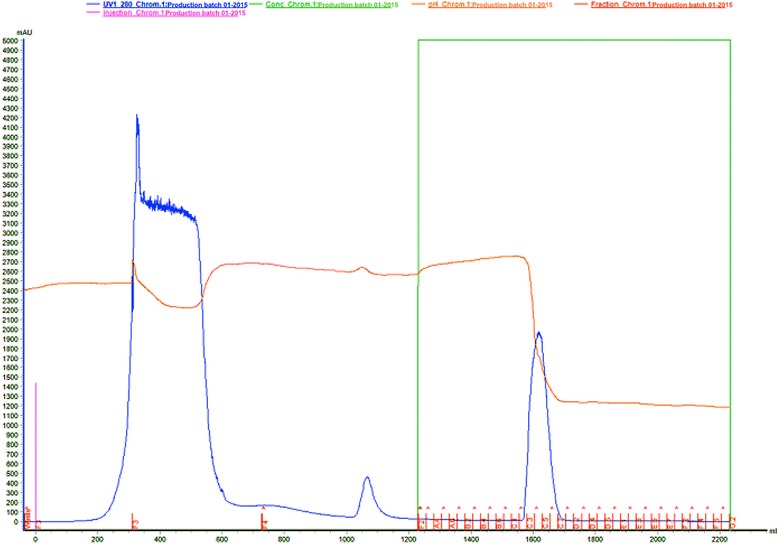
Affinity liquid chromatography of *Crotalus durissus terrificus* crude venom with Benzamidine-Sepharose 6B resin (column AxiChrom 100/300® (100 mm × 300 mm × 350 mL). The column was equilibrated with 0.05 M Tris-HCl pH 7.4 (buffer 1). Sample: 15 g of crude venom was suspended in buffer 1. The sample elution was carried out with 525 mL of buffer 1 followed by 525 mL of 0.05 M Tris-HCl/0.5 M NaCl pH 7.4 (buffer 2) and 1,050 mL of 0.02 M glycine pH 3.2 (buffer 3), at flow rate 10 mL/min, and collected 25 mL/tube. Absorbance 280 nm

To isolate the serine protease (gyroxin), a low-pressure liquid chromatography system was employed, specifically the model Äkta Pilot**®** (GE HealthCare Life Science, Sweden) and the software Unicorn**®** 6.3 controlled the data acquisition. All reagents and salts utilized were of HPLC grade, and the Milli-Q water used was obtained in a Milipore® ultra-purifier (Fig. 5).

Fifteen grams of lyophilized venom from *Crotalus durissus terrificus* was suspended in 200 mL of the buffer 0.05 M Tris-HCl pH 7.4. This was applied in an AxiChrom 100/300® chromatographic column (100 mm x 300 mm x 350 mL) (GE HealthCare Life Science, Sweden) encased with affinity resin Benzamidine Sepharose 6B® (GE HealthCare Life Science, Sweden) previously equilibrated with 525 mL of 0.05 M Tris-HCl pH 7.4 buffer (buffer 1). The sample was eluted with 525 mL of 0.05 M Tris-HCl pH 7.4 (buffer 1), followed by 525 mL of 0.05 M Tris-HCl/0.5 M NaCl pH 7.4 (Buffer 2) and 1,050 mL of glycine 0.02 M pH 3.2 (buffer 3). The flow utilized was 10 mL/min and collected 25 mL/tube. The elution was monitored at an absorbance of 280 nm.

This purification process generates a single fraction whose purity is evaluated by N-terminal sequences (EDMAN) and mass spectrometry.

**Fig. 6:**
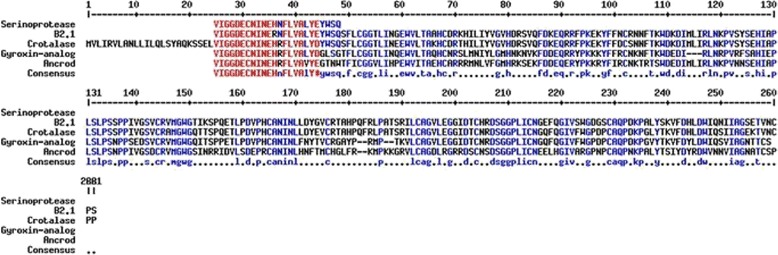
Comparison between the N-terminal sequences by multiple alignment by MultiAlin program. The first sequence shows the serine protease purified in this study, followed by the sequences deposited in the NCBI such as: B2.1 (thrombin-like enzyme from *Crotalus durissus terrificus* venom); crotalase (thrombin-like enzyme from *Crotalus adamanteus* venom); gyroxin analog (thrombin-like enzye from *Lachesis muta muta* venom) and ancrod (thrombin-like enzyme from *Agkistrodon rhodostoma*). The *red* letters indicate high similarity (90%) and *blue* a low similarity level (50%). The *black* letters indicate no similarity

**Fig. 7:**
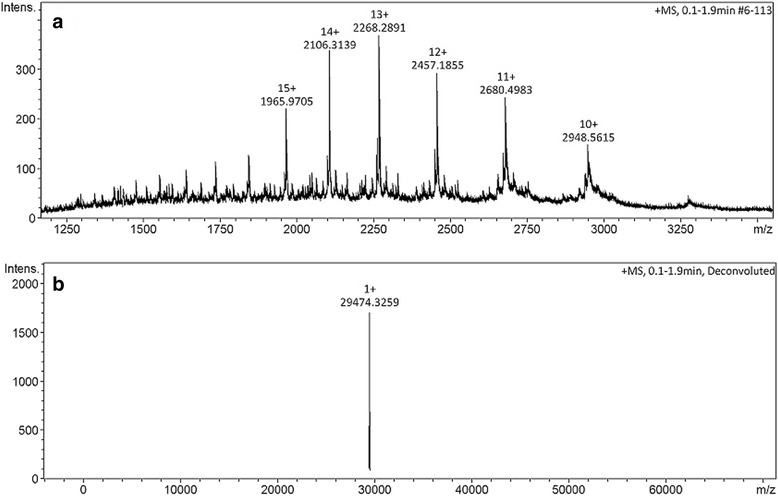
LC-MS by ESI-ToF mass spectra (MicroQ-ToF III, Bruker Daltonics®). **a** Different protonated forms of gyroxin from *Crotalus durissus terrificus*. **b** Deconvoluted mass spectrum showing [M + H]^+1^ = 29.472 m/z. Molecular mass of this serine protease is 29.473 Da

Figures 6 and 7 show, respectively, the comparison of N-terminal sequence of gyroxin with other thrombin-like snake toxins and their molecular mass by ESI mass spectrometry.

#### Biological activity

In 2011, Barros et al*.* [9, 18] evaluated the coagulant activity of a serine protease isolated from *Crotalus durissus terrificus* venom, which was able to induce the formation of a fibrin network and consequently the formation of a stable clot at different concentrations.

**Fig. 8:**
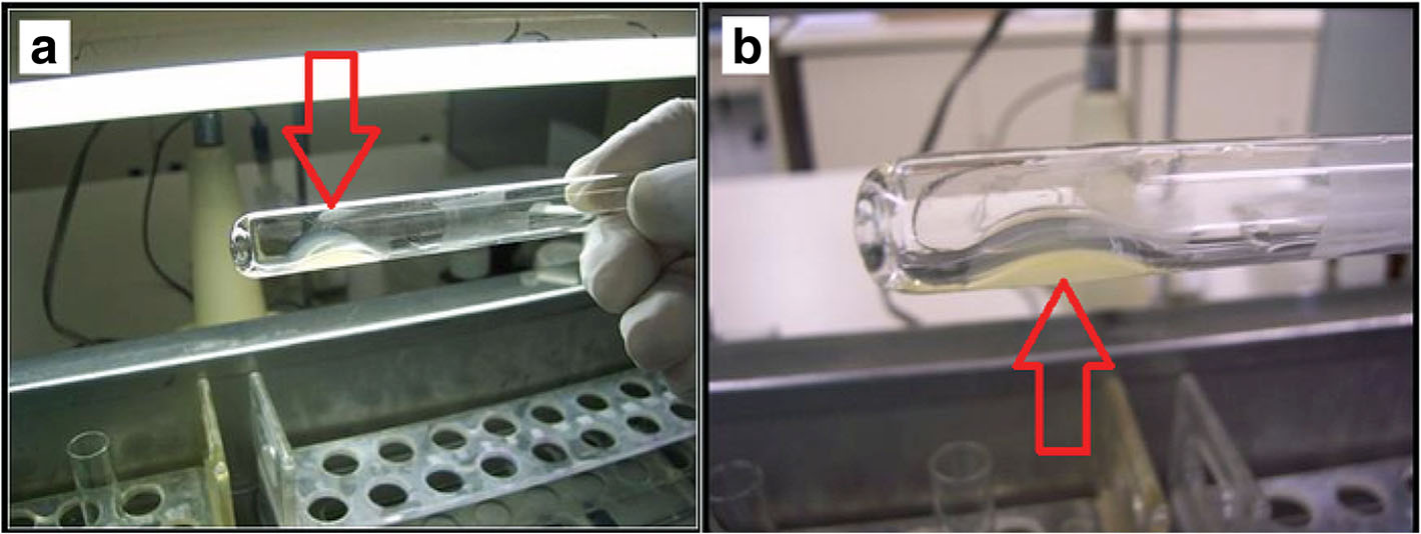
Coagulant activity: **a** fibrin clot formed after incubation of human plasma with serine protease; **b** detail of fibrin network

Coagulant activity was studied at three different pH, namely: 4.0, 6.0 and 7.4. At each of them, the minimum coagulant dose (MCD) was verified and defined as the quantity at which a certain enzyme is capable of coagulating 200 μL of plasma in 60 s [15]. At pH 4.0, the MCD was 0.037 μg/μL of plasma, versus 0.015 μg/μL at pH 6.0 and 0.021 μg/μL at pH 7.4. Table 1 and Fig. 8 display the MCD at pH 7.4.

**Table 1:** Serine protease concentrations employed to evaluate the clotting time, the mean of three measures, the standard deviation and standard error of the mean for a 95% confidence interval at pH 7.4

Concentration (μg)	Mean (seconds)	Standard deviation	Standard error of the mean
3	84	±3.60	2.08
5	50.7	±0.57	0.33
10	32.7	±0.57	0.33
15	20	±0.0	0.0
20	14.7	±0.57	0.33
25	13.3	±0.57	0.33

**Fig. 9:**
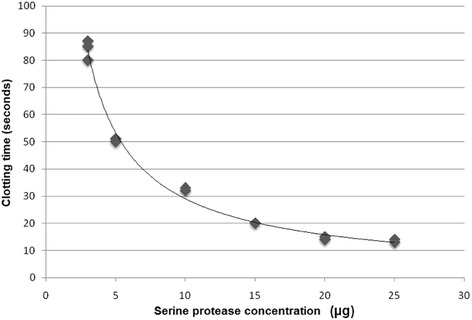
Evaluation at pH 7.4 of the minimum coagulant dose (MCD) of the serine protease by potential regression analysis y = 220.13x^-0,879^, *R* ^2^ = 0.9899

The serine protease coagulant activity at pH 7.4 was also confirmed through dose-dependent activity evaluated to obtain the MCD. For potential regression analysis, the MCD was determined at 0.021 μg/μL of human plasma, as shown in Fig. 9.

It must be emphasized that the statistical analysis did not present a difference in the comparison of serine protease activity at pH 6.0 versus pH 7.4. These results lead to the conclusion that the best activity of the enzyme is found at between pH 6 and 7.4, values close to the optimum pH for blood-thrombin activity, which is 7.3 and varies in blood between 7.35 and 7.45 [18].

### Fraction II: cryoprecipitate

Cryoprecipitate is the insoluble fraction, a cold precipitate of frozen fresh plasma (FFP) from *Bubalus bubalis* buffalos. It contains fibrinogen, factor VIII (F VIII), Willebrand factor (F vW), factor XIII (F XIII) and fibronectin [19, 20]. It must contain at least 80 units of factor VIII and between 150 and 250 mg of fibrinogen. Each unit has a volume from 10 to 20 mL, which must be stored at –20 ° C and has a shelf life of one year.

In 1995, Iuan et al. [5] proposed for the first time a new fibrin sealant constituted of a serine protease extracted from the venom of *Crotalus durissus terrificus* and human cryoprecipitate. The new product was compared with the commercial sealant in relation to the repair of sciatic nerves in Wistar rats. The anatomopathological analyses found similar results between the two products. Aiming at preventing infectious diseases transmitted by human blood, Thomazini-Santos et al*.* [21] in 1998 proposed for the first time to replace fibrinogen extracted from human blood with that from buffaloes. These same authors [21] evaluated the cryoprecipitate level of diverse animals and compared them with that extracted from human blood. They observed that buffaloes presented the highest fibrinogen levels, as shown in Table 2. Due to the good performance of the cryoprecipitate extracted from buffalos, these animals have been selected as the ideal donors.

**Table 2:** Comparison of fibrinogen concentration in mg% in human, bovine, equine, ovine and buffalo blood

Mean fibrinogen concentration in cryoprecipitate (mg%)				
Human (*N* = 12) G1	Bovine (*N* = 9) G2	Equine (*N* = 10) G3	Ovine (*N* = 10) G4	Buffalo (*N* = 7) G5
375.50 ± 70.95	218.33 ± 9.76	240.80 ± 72.03	267.70 ± 25.42	664.00 ± 11.96

Statistics: G1 × G2 × G3 × G4 × G5, F = 120.26, *p* < 0.001, G5 > G1 > (G2 = G3 = G4)

In Brazil, the following four buffalo breeds are recognized by the Brazilian Association of Buffalo Breeders: *mediterrâneo*, *murrah*, *jafarabadi* (river buffalo) and *carabao* (swamp buffalo). The *murrah* breed, *Bubalus bubalis*, of Indian origin has been raised at the Lageado Experimental Farm, UNESP campus in Botucatu, for more than 30 years [22].

In order to ensure that this bioproduct contains no substance foreign to the human body, it is necessary to select and certify the donors. Therefore, sanitary management is mandatory for good economic results, which includes the following actions: annual vaccination against foot-and-mouth disease, brucellosis and rabies; systematic deworming; measures for hygiene and asepsis; practices of isolation and quarantine; protection of animals against vectors of transmissible diseases; diagnostic serological tests against brucellosis and leptospirosis; an annual hypersensitivity test against tuberculosis (tests of tuberculinization and of Mantoux or PPD); as well as frequent clinical exams performed by an experienced veterinary physician. These actions are recommended by the Department of Animal Health of the Secretariat for the Defense of Agribusiness and Livestock Raising in the Ministry of Agriculture, Livestock and Food Supply (MAPA) and by the World Health Organization, and are in continuous execution by the abovementioned team [23–29].

Despite all these precautions, these animals can still pose a risk to human health due to transmission of spongiform encephalopathies (TSEs), also known as prion diseases, or as “mad cow” [29]. They are fatal neurodegenerative diseases that include *scrapie* in sheep, a bovine spongiform encephalopathy (BSE) and Creutzfeldt-Jakob disease (CJD) in humans. In buffalos, the transmission can occur through the consumption of previously infected tissue that is used in the feed manufacturing, particularly nerve tissue. In suspicious cases, the necropsy becomes the highest priority, followed by anatomical-pathological analysis. Researchers at CEVAP in partnership with the Center for Stable Environmental Isotopes, in the Botucatu Biosciences Institute of UNESP, developed a globally pioneering technique of isotopic tracers based on the dosing of carbon isotopes (^13^C) and nitrogen (^15^N) in sheep and buffaloes [30, 31]. After its standardization, this technique was tested in animals of the abovementioned herd, showing an absence of animal protein ingestion, which indirectly indicates that the buffalo donors of cryoprecipitate were potentially free of mad cow disease.

The growing concern with the rapid identification and resolution of sanitary problems in livestock has increased the interest in the study of biomarkers. Recent research has shown that the quantification of acute-phase proteins in blood can offer useful information for early diagnosis, prognosis and monitoring of diseases [32]. These proteins are considered not only potential indicators of inflammatory disease or contagious infections, but also an important tool in slaughterhouses to ensure food safety [33, 34].

The concentrations of these proteins, which remain circulating for long periods, depend on the severity of the dysfunction. Therefore, their quantification is an essential tool to evaluate the presence and severity of the inflammatory process, in contrast to the cytokines that remain circulating for short periods and whose measurement is onerous [35].

The first response of the organism to immunological stress is a non-specific release of cytokines that are mediators in the variation of acute-phase proteins [34, 35]. Through the influence of interleukins 1 and 6 (IL-1, IL-6) and of tumor necrosis factor (TNF-α), the hepatic cells augment or diminish the synthesis and the secretion of certain proteins. The response occurs immediately after a lesion or disease, declining within one or two days. The acute-phase proteins can be divided into two groups: negative and positive. The negative proteins are those that diminish the concentration when an acute-phase response occurs – and include albumin and transferrin, while the positive ones have their level elevated when there is an acute-phase response. In the latter case we have an increase in circulating C-reactive protein (CRP), glycoprotein-1 acid, antitrypsin-1, antichemotrypsin-1, serum amyloid A, ceruloplasmin, haptoglobin, macroglobin-2, fibrinogen and component C_3_ of the complement system [34, 36, 37].

For ruminants, haptoglobin has been described as the most important and reliable marker [32, 33]. Thus, the standardization of acute-phase biomarkers (fibrinogen and haptoglobin) and the clinical evaluation prior to blood donation permit the presumptive diagnosis of possible diseases and the removal of the donor animal to ensure the extraction of a safe bioproduct.

A rigorous protocol was applied in order to maintain biosecurity and the traceability of cryoprecipitate, as follows:

- In the herd of buffalos:
  - ➢ microchipping permitting traceability *a posteriori*;
  - ➢ annual vaccination control against rabies, brucellosis and foot-and-month disease;
  - ➢ application and annual evaluation of the tuberculin;
  - ➢ control of spongiform encephalopathy (mad cow disease) by means of isotopic analysis;
  - ➢ nonspecific presumptive diagnosis of diseases for selection of ideal donors by means of haptoglobin and fibrinogen biomarkers.
- In blood collection:
  - ➢ utilizing quadruple bag with filters in a line similar to those employed for humans;
  - ➢ transporting the bags with blood in refrigerated boxes to the processing laboratory;
  - ➢ applying techniques to evaluate fibrinogen levels and the factors V, VIII and von Willebrand;
  - ➢ preventing possible contaminations of the bags utilizing for quality control animal blood culture and of the bags in Bactec**®** for aerobic and anaerobic bacteria and Bactec Myco-F Lytic**®** for fungi.

**Fig. 10:**
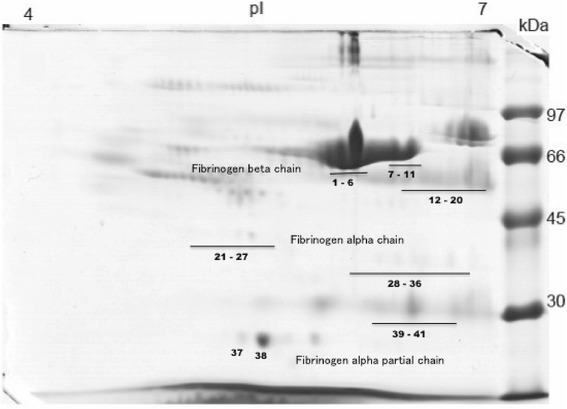
*Bubalus bubalis* cryoprecipitate protein profile (2D-SDS-PAGE) showing 40 different forms of fibrinogen molecules

Finally, analytical methods with singular characteristics, such as higher sensitivity, resolution and reproducibility were employed with a clinical proteomic approach [38]. Two-dimensional electrophoresis (2D) was used for isolating and identifying proteins by means of their molecular masses and isoelectric points in polyacrylamide gel, and electrospray-type mass spectrometry was used for sequencing peptides and proteins and identifying their biological function. Figure 10 shows the total protein profile of cryoprecipitate extracted from buffalos presenting the different forms of fibrinogen, evidenced for better visualization, since this protein is the main molecule responsible for stable fibrin clot formation. There are three observable classes of fibrinogen denominated: β-chain fibrinogen, from α and partial forms of α-chain fibrinogen, totaling 40 different forms of the molecule.

In sum, the cryoprecipitate extracted to be applied as a new heterologous fibrin sealant standardized by CEVAP is a product that is safe and free from undesirable substances. The formulation, as well as its storage, handling and dosage are described in detail in the internationally required patents (PCT/BR2015/000065 and PCT/BR2015/000064) [39].

## The human use of heterologous fibrin sealant

The heterologous fibrin sealant, widely studied experimentally, is now in a phase I/II clinical trial for the treatment of chronic venous ulcers. Herein, we briefly describe the methodology utilized for manufacturing this new biomedicine that possesses vast potential to replace the human constituents utilized in commercial sealants currently available on the market. This product has undergone more than 20 years of development and due to its novelty and originality, it represents a success story in the context of World Toxinology, mostly in the southern hemisphere.

Until the present, two phase I/II clinical trials (called Sealant I and Sealant II) have been proposed for evaluating the treatment of chronic venous ulcers. For this purpose, four batches of sealant were produced for application in ten participants in the first project already concluded (Sealant I) and, in 30 participants in the second, which is now underway (Sealant II).

For the determination of the protein concentration of serine protease (gyroxin) utilized in the finished product, protein dosing was performed via direct reading at 280 nm utilizing a NanoView® spectrophotometer (GE Healthcare, USA). This apparatus quantifies the concentration of proteins according to the law of Lambert-Beer [40]. In this manner, the gyroxin quantity sufficient for polymerizing the fibrin contained in 1 mL of cryoprecipitate was defined for each 2 mL-dose of fibrin sealant. This quantity of polymer should cover an ulcer with a maximum size of 60 cm^2^. A 1 mL vial of cryoprecipitate contains, in addition to fibrinogen, the following coagulation factors: factor V, VIII and von Willebrand. The diluent vial contains 0.6 mL of a stable solution of calcium chloride. The details of this composition are described in the submitted patents [39].

**Fig. 11:**
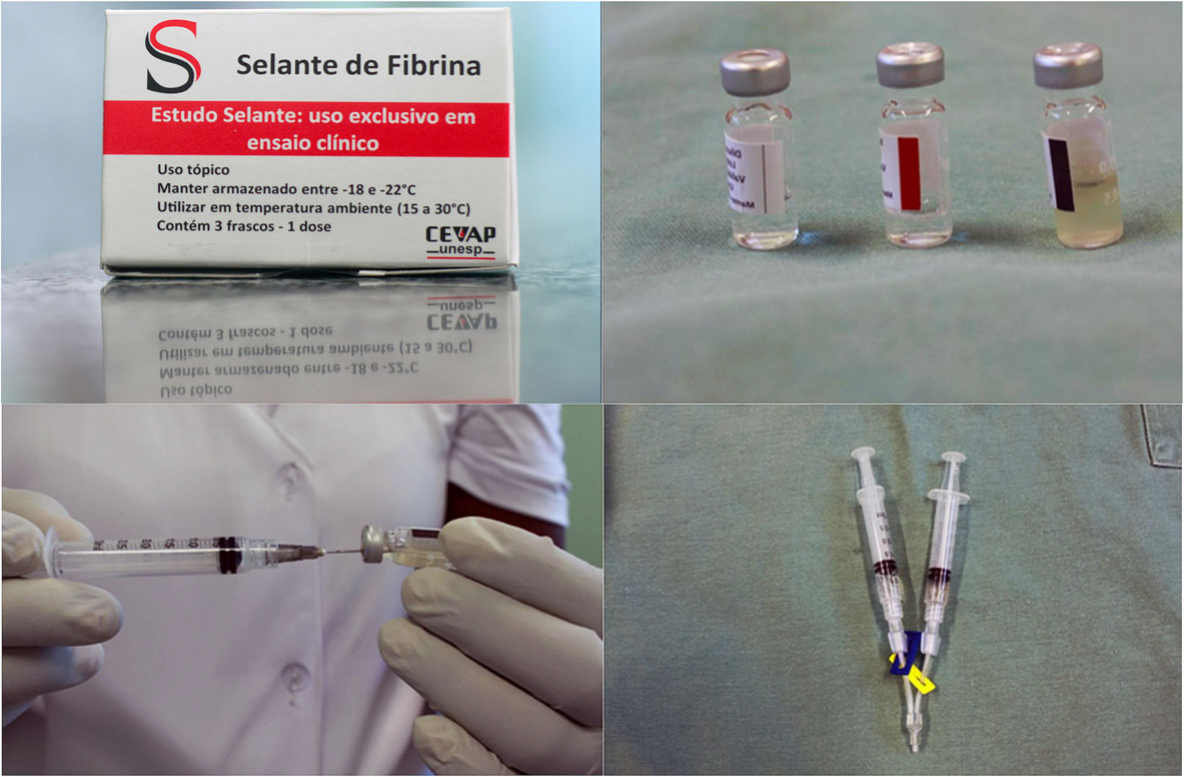
Packaging and vials approved only for clinical research use

**Fig. 12:**
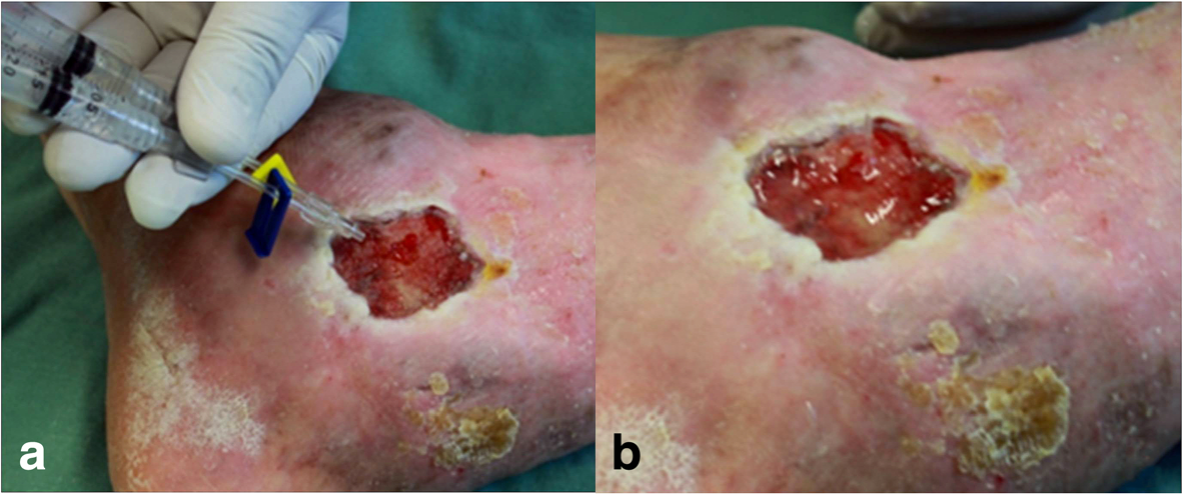
**a** Application of the product utilizing a double-outlet syringe with mixer at its end. **b** Polymerized product covering an ulcer

Figures 11 and 12 demonstrate the product packaged for clinical research, with attention to the protocols of the National Health Surveillance Agency (ANVISA) in Brazil, as well as its preparation for application in ulcers of the participants (ethics approval CONEP-CAAE: 19006813.4.1001.5411).

The objectives of the Sealant I project were already achieved, namely: to study safety and most appropriate dose of the new heterologous fibrin sealant for treating chronic venous ulcers.

**Fig. 13:**
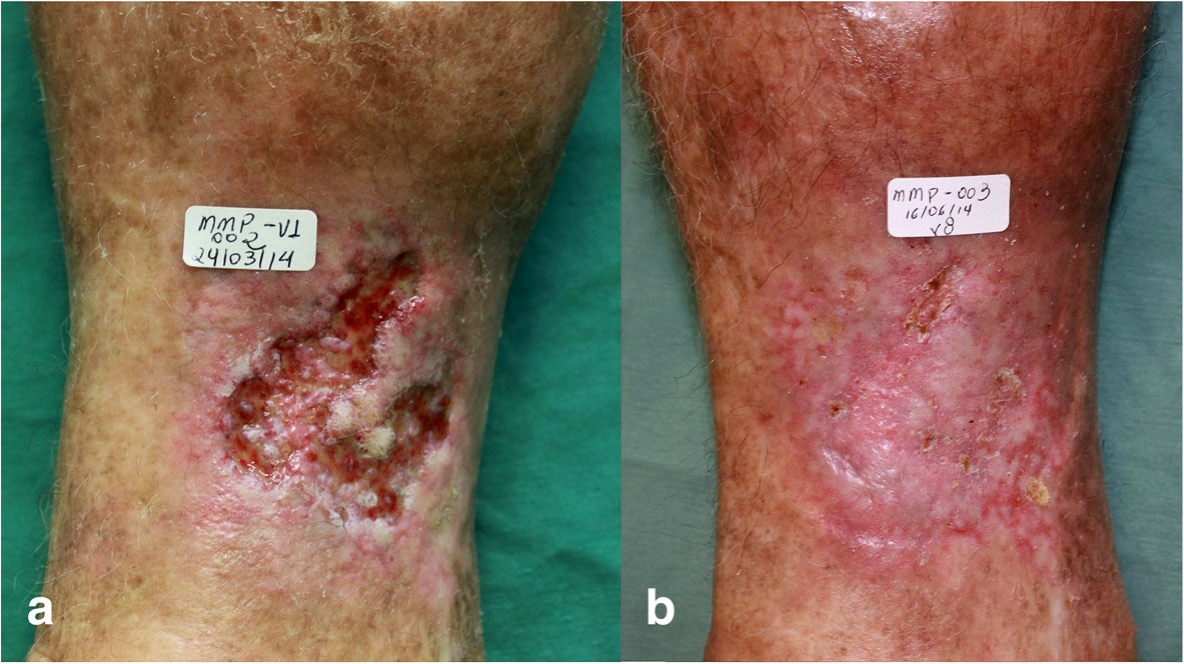
A 70 year-old female had an ulcer for two years. **a** Visit 0 – area of the ulcer was 17.1 cm^2^. **b** Visit 6 – wound healed

**Fig. 14:**
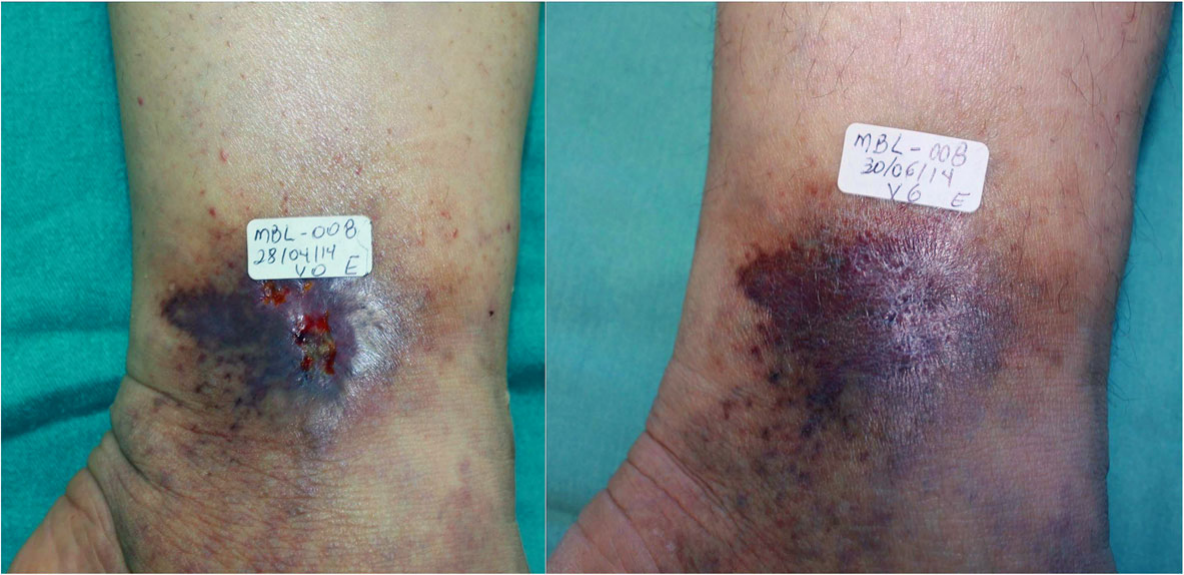
The 50 year-old patient had an ulcer for 4 months. **a** Visit 0 – area of the ulcer was 0.3 cm^2^. **b** Visit 6 – wound healed

Figures 13 and 14 show the evolution and ulcers healing in two participants, before (V0) and at the end of treatment.

The new heterologous fibrin sealant is a safe and clinically promising candidate for treating chronic venous ulcers. A multicenter clinical trial, phase II/III, with a larger number of participants will be performed to prove the efficacy of the product [41]. A six-minute video is provided showing an overview of the production and application of the fibrin sealant (Additional file 1) (available at: https://youtu.be/y6ho6M0amA8).

## Conclusions

The homologous commercial fibrin sealant has been used with success since the 1970s. Nowadays, its application has been consolidated in surgical procedures as an efficient method to avoid suturing, decrease recovery time and increase the success rate. Its indications are well defined and proven through a systematic review of studies and meta-analysis [42–45]. Among the unsolved problems, especially in biopharmaceutical production, are the high costs and transmission of infectious diseases by human blood [3, 4]. The new heterologous fibrin sealant, composed primarily of extracted animal products, has low production costs and does not transmit infectious diseases. Standardized for over 20 years by a consortium of Brazilian researchers, several preclinical studies and clinical trials have already been completed. Thus, preclinical trials applying the product in the peripheral nervous and musculoskeletal systems [46–56] and as a scaffold for stem cells have been studied extensively [57–60]. Trials in plastic surgery skin repair [61], periodontal surgery [62–64] and in chronic venous ulcers have also been performed [41, 65]. In addition to treating chronic venous ulcers, further clinical trials, especially ones linked to the nervous system and to skeletal muscle, will allow for a more precise use.

## Additional file

Additional file 1: The video shows a six-min overview of the production and application of the fibrin sealant derived from snake venom and buffalo blood (available at https://youtu.be/y6ho6M0amA8). (DOCX 11 kb)

## Abbreviations

2D: Two-dimensional electrophoresis
ANVISA: National Health Surveillance Agency
BSE: Bovine spongiform encephalopathy
CEVAP: Center for the Study of Venoms and Venomous Animals
CJD: Creutzfeldt-Jakob disease ()
CRP: C-reactive protein
FDA: U.S. Food and Drug Administration
FFP: Frozen fresh plasma
GMP: Good manufacturing practices
HFS: Heterologous fibrin sealant
IL-1: Interleukin 1
IL-6: Interleukin 6
MCD: Minimum coagulant dose
TNF: Tumor necrosis factor
TSEs: Transmission of spongiform encephalopathies

## References

[1] Spotnitz WD, Burks S (2012). Hemostats, sealants, and adhesives III: a new update as well as cost and regulatory considerations for components of the surgical toolbox. Transfusion.

[2] Spotnitz WD (2014). Fibrin sealant: The only approved hemostat, sealant, and adhesive — a laboratory and clinical perspective. ISRN Surg.

[3] Hino M, Ishiko O, Honda KI, Yamane T, Ohta K, Takubo T (2000). Transmission of symptomatic parvovirus B19 infection by fibrin sealant used during surgery. Br J Haematol.

[4] Kawamura M, Sawafuji M, Watanabe M, Horinouchi H, Kobayashi K (2002). Frequency of transmission of human parvovirus B19 infection by fibrin sealant used during thoracic surgery. Ann Thorac Surg.

[5] Iuan FC, Thomazini IA, Giannini MJM, Viterbo F, Toscano E, Moraes RA (1995). Reparation of peripheral nerves with fibrin glue prepared from snake venom: preliminary results. Sao Paulo Med J.

[6] Biscola NP, Cartarozzi LP, Ulian-Benitez S, Barbizan R, Castro MV, Spejo AB (2017). Multiple uses of fibrin sealant for nervous system treatment following injury and disease. J Venom Anim Toxins incl Trop Dis.

[7] Thomazini-Santos IA (2001). Fibrin adhesive from snake venom: the effect of adding eaminocaproic acid, tranexamic acid and aprotinin for coaptation of wound in rat skin incisions. J Venom Anim Toxins.

[8] Thomazini-Santos IA, Barraviera SRCS, Mendes-Giannini MJS, Barraviera B (2001). Surgical adhesives. J Venom Anim Toxins.

[9] Barros LC, Ferreira RS, Barraviera SRCS, Stolf HO, Thomazini-Santos IA, Mendes-Giannini MJS (2009). A new fibrin sealant from *Crotalus durissus terrificus* venom: applications in medicine. J Toxicol Environ Health B Crit Rev.

[10] Gasparotto VPO, Landim-Alvarenga FC, Oliveira ALR, Simões GF, Lima-Neto JF, Barraviera B (2014). A new fibrin sealant as a three-dimensional scaffold candidate for mesenchymal stem cells. Stem Cell Res Ther.

[11] Seki C, Vidal JC, Barrio A (1980). Purification of gyroxin from a South American rattlesnake (*Crotalus durissus terrificus)* venom. Toxicon.

[12] Raw I, Rocha MC, Esteves MI, Kamiguti AS (1986). Isolation and characterization of a thrombin-like enzyme from the venom of *Crotalus durissus terrificus*. Braz J Med Biol Res.

[13] Bercovici D, Chudziniski AM, Dias WO, Esteves MI, Hiraichi E, Oishi NY (1987). A systemic fractionation of *Crotalus durissus terrificus* venom. Mem Inst Butantan.

[14] Rawling ND, Tolle DP, Barret AJ (2004). Evolutionary families of peptidase inhititors. Biochem J.

[15] Andrews RK, Gardiner EE, Berndt MC (2004). Snake venom toxins affecting platelet function. Methods Mol Biol.

[16] de Oliveira DGL, Murakami MT, Cintra ACO, Franco JJ, Sampaio SV, Arni RK (2009). Functional and structural analysis of two fibrinogen-activating enzymes isolated from the venoms of *Crotalus durissus terrificus* and *Crotalus durissus collilineatus*. Acta Biochim Biophys Sin Shanghai.

[17] Buchi AT (2010). Purification, characterization, crystallization and theoretical molecular modeling of gyroxin fraction from *Crotalus durissus terrificus* venom (Laurenti, 1768). J Venom Anim Toxins incl Trop Dis.

[18] Barros LC, Soares AM, Costa FL, Rodrigues VM, Fuly AL, Giglio JR (2011). Biochemical and biological evaluation of gyroxin isolated from *Crotalus durissus terrificus* venom. J Venom Anim Toxins incl Trop Dis.

[19] O’Shaughnessy DF, Atterbury C, Bolton Maggs P, Murphy M, Thomas D, Yates S (2004). Guidelines for the use of fresh-frozen plasma, cryoprecipitate and cryosupernatant. Br J Haematol.

[20] Nascimento B, Goodnough LT, Levy JH (2014). Cryoprecipitate therapy. Br J Anaesth.

[21] Thomazini-Santos IA, Giannini MJSM, Toscano E, Machado PEA, Lima CRG, Barraviera B (1998). The evaluation of clotting time in bovine thrombin, Reptilase^®^, and thrombin-like fraction of *Crotalus durissus terrificus* venom using bovine, equine, ovine bubaline and human cryoprecipitates. J Venom Anim Toxins.

[22] Associação Brasileira de Criadores de Búfalos. Available in 10 Aug 2016 at http://www.bufalo.com.br/home/

[23] Ministério da Agricultura, Pecuária e Abastecimento. Plano de ação para febre aftosa. Ministério da Agricultura, Pecuária e Abastecimento. Secretaria de Defesa Agropecuária - Brasília: MAPA/SDA/DAS; 2009. p. 96.

[24] Ministério da Agricultura, Pecuária e Abastecimento. Programa Nacional de Controle e Erradicação da Brucelose e da Tuberculose Animal (PNCEBT), Brasília: MAPA/SDA/DAS; 2006. p. 188.

[25] Ministério da Agricultura, Pecuária e Abastecimento Controle da Raiva dos herbívoros – Brasília; 2005. p. 104.

[26] Barros CSL, Marques GHF. Procedimentos para o diagnóstico das doenças do sistema nervoso central de bovinos. Brasília; 2003. p. 50.

[27] Ministério da Agricultura, Pecuária e Abastecimento (2009). Manual de Legislação: programas nacionais de saúde animal do Brasil. Ministério da Agricultura, Pecuária e Abastecimento. Secretaria de Defesa Agropecuária.

[28] Manual de procedimentos para a atenção às ocorrências de febre aftosa e outras enfermidades vesiculares. Projeto BID/PANAFTOSA - OPAS/OMS para os países do MERCOSUL Ampliado. Rio de Janeiro: PANAFTOSA - OPAS/OMS; 2007. p. 144.

[29] Zoonoses and veterinary public health. World Health Organization. Available in 10 Aug 2016 at http://www.who.int/zoonoses/diseases/prion_diseases/en/

[30] da Silva DA F, Biscola NP, Souza RMF, Caetano DA, Denadai JC, Sartori MMP (2012). Carbon-13 and Nitrogen-15 turnover in serum of bubaline donors of biological material for medical use. Toxicon.

[31] Fossato da Silva D, Biscola NP, dos Santos LD, Sartori MMP, Denadai JC, Silva ET (2016). Detecting animal by-product intake using stable isotope ratio mass spectrometry (IRMS). Vet J.

[32] Petersen HH, Nielsen JP, Heegaard PMH (2004). Application of acute phase protein measurements in veterinary clinical chemistry. Vet Res.

[33] Petersen HH, Ersbøll AK, Jensen CS, Nielsen JP (2002). Serum-haptoglobin concentration in Danish slaughter pigs of different health status. Prev Vet Med.

[34] Gruys E, Toussaint MJM, Niewold TA, Koopmans SJ (2005). Acute phase reaction and acute phase proteins. J Zhejiang Univ Sci B.

[35] Gutierrez AM, Martinez-Subiela S, Eckersall PD, Cerón JJ (2009). C-reactive protein quantification in porcine saliva: a minimally invasive test for pig health monitoring. Vet J.

[36] Ferreira RS, Almeida RAMB, Barraviera SRCS, Barraviera B (2012). Historical perspective and human consequences of africanized bee stings in the Americas. J Toxicol Environ Health B Crit Rev.

[37] Barraviera B, Lomonte B, Tarkowski A, Hanson LA, Meira DA (1995). Acute-phase reactions, including cytokines, in patients bitten by *Bothrops* and *Crotalus* snakes in Brazil. J Venom Anim Toxins.

[38] Tabb DL (2013). Quality assessment for clinical proteomics. Clin Biochem.

[39] Ferreira RS (2014). Autologous or heterologous fibrin sealant scaffold: which is the better choice?. J Venom Anim Toxins incl Trop Dis.

[40] Mäntele W, Deniz E (2017). UV–VIS absorption spectroscopy: Lambert-Beer reloaded. Spectrochim Acta A Mol Biomol Spectrosc.

[41] Abbade LPF, Barraviera SRCS, Silvares MRC, Carneiro MTR, Medolago NB, Ferreira RS (2015). A new fibrin sealant derived from snake venom candidate to treat chronic venous ulcers. J Am Acad Dermatol.

[42] Li J, Li HB, Zhai XC, Qin-Lei, Jiang XQ, Zhang ZH (2016). Topical use of topical fibrin sealant can reduce the need for transfusion, total blood loss and the volume of drainage in total knee and hip arthroplasty: A systematic review and meta-analysis of 1489 patients. Int J Surg.

[43] Rogers AC, Turley LP, Cross KS, McMonagle MP (2016). Meta-analysis of the use of surgical sealants for suture-hole bleeding in arterial anastomoses. Br J Surg.

[44] Brustia R, Granger B, Scatton O (2016). An update on topical haemostatic agents in liver surgery: systematic review and meta analysis. J Hepatobiliary Pancreat Sci.

[45] Weldrick C, Bashar K, O’Sullivan TA, Gillis E, Clarke Moloney M, Tang TY (2014). A comparison of fibrin sealant versus standard closure in the reduction of postoperative morbidity after groin dissection: A systematic review and meta-analysis. Eur J Surg Oncol.

[46] Barbizan R, Castro MV, Rodrigues AC, Barraviera B, Ferreira RS, Oliveira ALR (2013). Motor recovery and synaptic preservation after ventral root avulsion and repair with a fibrin sealant derived from snake venom. PLoS One.

[47] Spejo AB, Carvalho JL, Goes AM, Oliveira AL (2013). Neuroprotective effects of mesenchymal stem cells on spinal motoneurons following ventral root axotomy: synapse stability and axonal regeneration. Neuroscience.

[48] Benitez SU, Barbizan R, Spejo AB, Ferreira RS, Barraviera B, Goes AM (2014). Synaptic plasticity and sensory-motor improvement following fibrin sealant dorsal root reimplantation and mononuclear cell therapy. Front Neuroanat.

[49] Barbizan R, Castro MV, Barraviera B, Ferreira RS, Oliveira ALR (2014). Influence of delivery method on neuroprotection by bone marrow mononuclear cell therapy following ventral root reimplantation with fibrin sealant. PLoS One.

[50] Barbizan R, Castro MV, Ferreira Junior RS, Barraviera B, Oliveira ALR (2014). Long-term spinal ventral root reimplantation, but not bone marrow mononuclear cell treatment, positively influences ultrastructural synapse recovery and motor axonal regrowth. Int J Mol Sci.

[51] Buchaim RL, Andreo JC, Barraviera B, Ferreira RS, Buchaim DV, Rosa GM (2015). Effect of low-level laser therapy (LLLT) on peripheral nerve regeneration using fibrin glue derived from snake venom. Injury.

[52] Castro MV, Barbizan R, Ferreira RS, Barraviera B, Oliveira ALR (2016). Direct spinal ventral root repair following avulsion: effectiveness of a new heterologous fibrin sealant on motoneuron survival and regeneration. Neural Plast.

[53] Biscola NP, Cartarozzi LP, Ferreira RS, Barraviera B, Oliveira ALR (2016). Long-standing motor and sensory recovery following acute fibrin sealant based neonatal sciatic nerve repair. Neural Plast.

[54] Buchaim DV, Rodrigues A de C, Buchaim RL, Barraviera B, Ferreira Jr RS, Jr GM (2016). The new heterologous fibrin sealant in combination with low-level laser therapy (LLLT) in the repair of the buccal branch of the facial nerve. Lasers Med Sci.

[55] de Oliveira Gonçalves JB, Buchaim DV, de Souza Bueno CR, Pomini KT, Barraviera B, Ferreira RS (2016). Effects of low-level laser therapy on autogenous bone graft stabilized with a new heterologous fibrin sealant. J Photochem Photobiol B.

[56] Spejo AB, Chiarotto GB, Ferreira Jr RS, Barraviera B, Oliveira ALR. Effects of mesenchymal stem cell and fibrin sealant treatment following spinal cord injury. 12th International Congress of Cell Biology (ICCB 2016), Vol. 1, Praga, República Tcheca; 2016. p.1-3

[57] de Barros CN, Miluzzi Yamada AL, Ferreira RS, Barraviera B, Hussni CA, de Souza JB (2016). A new heterologous fibrin sealant as a scaffold to cartilage repair - Experimental study and preliminary results. Exp Biol Med (Maywood).

[58] Machado EG, Issa JPM, Figueiredo FAT, Santos GR, Galdeano EA, Alves MC (2015). A new heterologous fibrin sealant as scaffold to recombinant human bone morphogenetic protein-2 (rhBMP-2) and natural latex proteins for the repair of tibial bone defects. Acta Histochem.

[59] Cunha MR, Menezes FA, Santos GR, Pinto CAL, Barraviera B, Martins VCA (2015). Hydroxyapatite and a new fibrin sealant derived from snake venom as scaffold to treatment of cranial defects in rats. Mat Res.

[60] Cartarozzi LP, Spejo AB, Ferreira RS, Barraviera B, Duek D, Carvalho JL (2015). Mesenchymal stem cells engrafted in a fibrin scaffold stimulate Schwann cell reactivity and axonal regeneration following sciatic nerve tubulization. Brain Res Bull.

[61] Stolf HO (1999). The use of fibrin adhesive derived from snake venom and the evaluation of skin grafting using skin from the patient’s nasolabial fold. J Venom Anim Toxins.

[62] Barbosa MDS, Greghi SLA, Passanezi E (2007). Fibrin adhesive derived from snake venom in periodontal surgery. J Periodontol.

[63] Barbosa MD, Stipp AC, Passanezi E, Greghi SL (2008). Fibrin adhesive derived from snake venom in periodontal surgery: histological analysis. J Appl Oral Sci.

[64] Chiquito GCM (2007). Comparison between suture and fibrin adhesive derived from snake venom for fixation of connective tissue graft in correction of marginal tissue recession. J Venom Anim Toxins incl Trop Dis.

[65] Gatti MAN, Vieira LM, Barraviera B, Barraviera SRCS (2011). Treatment of venous ulcers with fibrin sealant derived from snake venom. J Venom Anim Toxins incl Trop Dis.

