# Supplementary material for: Heterologous fibrin sealant derived from snake venom: from bench to bedside – an overview
Source: J Venom Anim Toxins Incl Trop Dis. 2017 Apr 4;23:21. doi: 10.1186/s40409-017-0109-8 (PMC5379742; doi:10.1186/s40409-017-0109-8)
Supplement: Additional file 1: — The video shows a six-min overview of the production and application of the fibrin sealant derived from snake venom and buffalo blood (available at https://youtu.be/y6ho6M0amA8). (DOCX 11 kb) [file 40409_2017_109_MOESM1_ESM.docx]

**Youtube video:** <https://youtu.be/y6ho6M0amA8>

**Additional file 1.** The video shows a 6-minute overview of the production and application of the fibrin sealant derived from snake venom and buffalo blood (available at <https://youtu.be/y6ho6M0amA8>).
